# Integrated Effects of Water and Nitrogen Coupling on Eggplant Productivity, Fruit Quality, and Resource Use Efficiency in a Cold and Arid Environment

**DOI:** 10.3390/plants14020210

**Published:** 2025-01-13

**Authors:** Jie Li, Hengjia Zhang, Chenli Zhou, Anguo Teng, Lian Lei, Yuchun Ba, Jiandong Yu, Fuqiang Li

**Affiliations:** 1 College of Agricultural and Biology, Liaocheng University, Liaocheng 252059, China; LJ824756@163.com (J.L.); zcl13830689195@163.com (C.Z.); 2 Yimin Irrigation Experimental Station, Hongshui River Management Office, Minle 734500, China; tenganguo@126.com (A.T.); ll4426072@163.com (L.L.); bayuchun@163.com (Y.B.); jiandongyu99@163.com (J.Y.); 3 College of Water Conservancy and Hydropower Engineering, Gansu Agricultural University, Lanzhou 730070, China; lifuq@gsau.edu.cn

**Keywords:** eggplant, water and nitrogen management, wa ter–nitrogen use efficiency, yield, quality

## Abstract

In order to explore the water and fertilizer requirements of eggplants in the western oasis of the river, the experiment was conducted in Minle County of Gansu Province in 2022 and 2023 under three water stress gradients and three nitrogen application levels: (1) moderate water stress (W_1_, 50–60% in field water capacity [FC]), mild water stress (W_2_, 60–70% in FC), and full irrigation (W_3_, 70–80% in FC); (2) low nitrogen (N_1_, 215 kg·ha^−1^), medium nitrogen (N_2_, 270 kg·ha^−1^), and high nitrogen (N_3_, 325 kg·ha^−1^). Moderate and mild water stress were applied during eggplant flowering and fruiting while full irrigation was provided during the other growth stages; a control class (CK) was established with full irrigation throughout the whole plant growth without nitrogen application. This study investigated the effects of water-saving and nitrogen reduction on the yield, quality, and water-nitrogen use efficiency of eggplants in a cold and arid environment in the Hexi Oasis irrigation area of China. Using the EWM-TOPSIS model, we evaluated different water-nitrogen treatments and determined the optimal irrigation-nitrogen application model for eggplants in this region. The results showed that the W_2_N_2_ treatment had the highest yield, which was not significantly (*p* > 0.05) different from the W_3_N_2_ treatment while significantly (*p* < 0.05) 35.06% higher than CK in 2022 and 36.91% higher in 2023. In the W_2_N_2_ treatment, the transverse diameter of eggplants, as well as the contents of soluble protein, soluble sugar, soluble solids, and vitamin C, were all the highest. The W_2_N_2_ treatment had the maximum water use efficiency and irrigation water use efficiency, which were significantly higher than other water and nitrogen application treatments and CK by 14.79–42.51% in 2022 and 8.79–44.88% in 2023, and 15.86–45.55% in 2022 and 4.68–40.22% in 2023, respectively. By employing the EWM-TOPSIS model for comprehensive evaluation, the results indicated that mild water deficit (60–70% in FC) and moderate nitrogen application (270 kg·ha^−1^) at flowering and fruiting of eggplants was the optimal water and nitrogen application mode under mulched drip irrigation in the Hexi region of northwest China. The results will provide some theoretical basis for water-saving, productive, high-quality, and high-efficiency cultivation of eggplant in cold and arid environments.

## 1. Introduction

Eggplant (*Solanum melongena* L.), a globally cultivated vegetable, is cherished for its nutritious flesh that is rich in protein, vitamins, and alkaloids [1], and its skin, which is abundant in anthocyanins and flavonoids [2], holds significant dietary and medicinal value. The Hexi region, capitalizing on its distinctive natural conditions, has established an agricultural production system predominantly based on irrigation agriculture. However, the expansion of oases and the increase in cultivated land have led to escalating water resource pressures in the Hexi region, posing a potential threat to the sustainable development of agriculture in the area [3]. Water serves as a critical medium for dissolving and transporting nutrients, thereby facilitating their absorption and transformation by the crop’s root system and playing a pivotal role in fertilizer efficacy. Nitrogen fertilizers, on the other hand, supply the necessary nutrients for crop growth and create conditions conducive to the efficient production of soil and water systems. Water and nitrogen fertilizers complement each other. Therefore, the study of scientific water and nitrogen management techniques and the determination of optimal irrigation and nitrogen application patterns can conserve water and nitrogen resources, mitigate environmental pollution, and provide a theoretical foundation for increasing eggplant yields and enhancing fruit quality.

Irrigation is a critical factor in ensuring the normal growth of crops. Proper irrigation not only improves soil physical properties, such as reducing soil compaction by increasing soil porosity [4] and promotes crop root expansion and nutrient uptake, but also reduces the frequency of crop pests and diseases and enhances their resilience to adversity by adjusting the microclimatic conditions in the field, such as by reducing temperature and humidity. However, irrational irrigation management may lead to problems such as flooding and soil salinization [5], and these may negatively affect crop growth. Nitrogen is the main nutrient element that affects crop growth, and once the amount of nitrogen fertilizer applied exceeds the appropriate range for the crop, it may cause problems such as soil nitrogen accumulation [6], which may negatively affect crop yield and quality. Qi et al. [7] found that a combination of 60–65% field capacity (FC) with 200–300 kg·ha^−1^ of nitrogen or 75–80% FC with 300 kg·ha^−1^ of nitrogen is an effective irrigation and nitrogen application pattern for achieving high grain yields in maize under the APRI (alternate partial root-zone irrigation) system in the northwestern corridor of China and similar environments. Adequate moisture conditions ensure normal physiological and metabolic activities in crops, promoting nutrient absorption and transport, thereby enhancing their dietary value and flavor experience. The application of nitrogen fertilizer aids in the development of green leaves and improves photosynthetic efficiency, but excessive nitrogen can cause crops to mature late and reduce fruit quality. Zhou et al. [8] found that under mild water deficit and moderate nitrogen application, eggplant fruits have the highest content of soluble protein, soluble solids, and vitamin C. Studies have shown that water-saving irrigation techniques such as drip irrigation can significantly improve water use efficiency (WUE) while ensuring crop yields. Moreover, moderate water deficit stimulates root growth and development, enhancing the plant’s water absorption capacity and further increasing WUE [9]. Appropriate nitrogen application positively affects crop growth and can effectively increase yields; however, excessive nitrogen not only reduces nitrogen use efficiency (NUE) but can also lead to nitrogen loss and environmental pollution. Water and nitrogen interact during crop growth, and some studies have found that integrated water and nitrogen management can significantly improve WUE and NUE in crops like corn and wheat [10,11].

At present, to comprehensively assess the relationships between fruit yield, quality, and water and nitrogen use efficiency, various methods have been proposed, including principal component analysis (PCA), gray correlation analysis (GRA), fuzzy comprehensive evaluation (FCE), and the technique for order preference by similarity to ideal solution (TOPSIS) method, each yielding different results [12]. The TOPSIS model is widely used to evaluate multiple indicators of crop production [13], but it has certain limitations in determining weights. It is susceptible to subjective influences, which can limit the objectivity and accuracy of the evaluation results. Therefore, this study introduces the entropy weight method (EWM) to obtain more reasonable weights. The EWM is an objective weighting method that reduces the impact of subjective factors and enhances the reliability of the evaluation results [14]. By using the EWM-TOPSIS model, we can effectively address multi-indicator evaluation problems. This model provides a comprehensive assessment of the effects of different irrigation and fertilization strategies on the yield, quality, and water and nitrogen use efficiency of eggplant, thereby identifying the optimal water and nitrogen management practices for this crop.

Currently, extensive research has been conducted on the impact of different irrigation and nitrogen applications on eggplant yield and quality [15,16,17]. However, there is relatively limited research on the comprehensive effects of the water–nitrogen interaction on eggplant, particularly in determining the optimal irrigation and nitrogen application strategies that can achieve a unified goal of water-saving, high yield, quality improvement, and high efficiency. This study posited that under challenging environmental conditions, an optimal synergy between water and nitrogen application exists that could be leveraged to enhance multiple aspects of eggplant cultivation. Specifically, we hypothesized that the best irrigation and nitrogen application strategy would not only improve the utilization efficiency of water and nitrogen in crops in the Hexi region but also elevate eggplant yield and quality. Therefore, the objectives of the present study were to determine the following: (1) the impact of the water–nitrogen interaction on eggplant yield, quality, and the water and nitrogen use efficiency under the cold and arid environment in the Hexi oasis region of northwest China; and (2) the optimal irrigation and nitrogen application pattern through a comprehensive evaluation using the EWM-TOPSIS model. Thus, the study was to provide a theoretical basis for high-yield and high-quality eggplant cultivation as well as highly efficient utilization of water resources and nitrogen fertilizer in a cold and arid environment.

## 2. Results

### 2.1. Water Consumption

#### 2.1.1. Water Consumption at Phasic Crop Growth Stages

**Figure 1:**
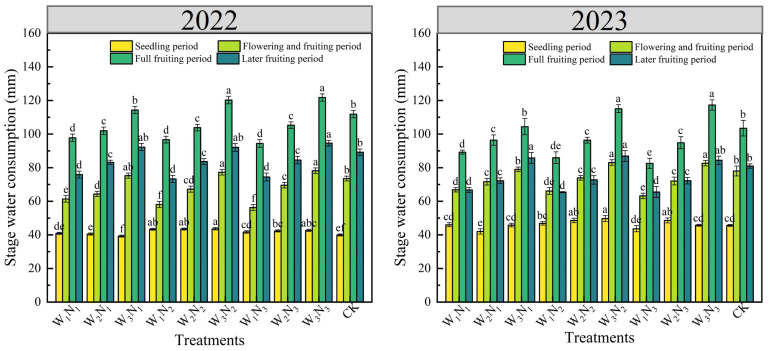
Effects of different water and nitrogen treatments on water consumption in the eggplant stage in 2022 and 2023. Different lowercase letters indicate significant differences between treatments at the *p* < 0.05 level. The bars represent the standard deviation.

The impact of different water and nitrogen treatments on eggplant water consumption during the two crop-growing seasons is shown in Figure 1. Water consumption at the seedling eggplant stage was relatively low, with the W_3_N_2_ treatment being observed to have the highest water consumption, which was significantly (*p* < 0.05) increased by 9.17% in 2022 and 8.83% in 2023 compared to the CK. At the flowering and fruiting stage, under water deficit treatment conditions, the highest water consumption was found in the W_3_N_3_ treatment in 2022 (78.14 mm) among all the treatments, with no significant difference (*p* > 0.05) compared to the W_3_N_1_ and W_3_N_2_ treatments while significantly increased by 6.31% compared to the CK and significantly increased by 12.38% to 38.99% compared to other water and nitrogen treatments. In 2023, the highest water consumption was observed in the W_3_N_2_ treatment (83.02 mm), with no significant difference compared to the W_3_N_3_ treatment, and it significantly increased by 6.44% compared to the CK and significantly increased by 5.10% to 31.36% compared to other water–nitrogen treatments. At the full fruiting stage, water consumption reached its peak; the highest water consumption was found in the W_3_N_3_ treatment, at 121.75 mm in 2022 and 117.31 mm in 2023, with no significant difference observed between this treatment and the W_3_N_2_ treatment, yet there was a significant increase of 8.87% in 2022 and 13.39% in 2023 when compared to the CK. At the later fruiting stage, water consumption gradually decreased. In 2022, the W_3_N_3_ treatment had the highest water consumption at 94.62 mm, significantly increased by 6.10% compared to the CK. In 2023, the W_3_N_2_ treatment had the highest water consumption at 86.88 mm, with a significant increase of 7.37% compared to the CK.

#### 2.1.2. Total Water Consumption

The total water consumption of eggplant ranged from 254.90 to 337.19 mm across water and nitrogen treatments in both growing seasons (Table 1). Both irrigation and nitrogen application had a highly significant (*p* < 0.01) effect on the total water consumption of eggplants, and their interaction also reached a significant (*p* < 0.05) level. In 2022, the W_3_N_3_ treatment exhibited the highest water consumption (337.19 mm), with no significant difference (*p* > 0.05) compared to the W_3_N_2_ treatment, while significantly (*p* < 0.05) increased by 7.24% compared to the CK and significantly increased by 5.04% to 24.24% compared to other treatments. In 2023, the W_3_N_2_ treatment had the highest water consumption (334.62 mm), slightly higher than the W_3_N_3_ treatment with no significant difference, and it significantly increased by 8.65% compared to the CK and significantly increased by 6.24% to 31.28% compared to other treatments. Under the same nitrogen application level, the total water consumption of eggplants decreased as the degree of water deficit increased. The W_1_ level significantly reduced water consumption by 5.09% to 26.42% (2022) and 5.01% to 29.49% (2023) compared to the W_2_ and W_3_ levels. The W_3_N_1_ treatment was at the same significance level as the CK, and it significantly decreased by 5.04% (2022) and 4.79% (2023) compared to the W_3_N_3_ treatment.

### 2.2. Yield

The eggplant yield across various water and nitrogen treatments in both growing seasons ranged from 58.81 to 87.87 t·ha^−1^ (Table 1). Irrigation, nitrogen application, and their interaction all had a highly significant (*p* < 0.01) effect on eggplant yield. The W_2_N_2_ treatment consistently yielded the highest, with no significant difference (*p* > 0.05) compared to the W_3_N_2_ treatment. In 2022, the W_2_N_2_ and W_3_N_2_ treatments significantly (*p* < 0.05) increased yield by 35.06% and 31.57%, respectively, compared to the CK; in 2023, the W_2_N_2_ and W_3_N_2_ treatments significantly increased yield by 36.91% and 34.49%, respectively, compared to the CK. The lowest yield was found in the W_1_N_3_ treatment in 2022 (58.81 t·ha^−1^), significantly reduced by 10.63% compared to the CK; in 2023, the W_1_N_3_ treatment had the lowest yield (61.38 t·ha^−1^), with no significant difference compared to the CK. Across both growing seasons, the mean total yield of eggplants under the same irrigation level was in the order of W_2_ > W_3_ > W_1_. In 2022, the W_2_ level significantly increased yield by 25.83% and 4.48% compared to the W_1_ and W_3_ levels, respectively; in 2023, the W_2_ level significantly increased yield by 22.90% and 4.62% compared to the W_1_ and W_3_ levels, respectively. Under the same nitrogen application level, the mean total yield of eggplants was in the order: N_2_ > N_3_ > N_1_. In 2022, the N_2_ level significantly increased yield by 14.95% and 11.26% compared to the N_1_ and N_3_ levels, respectively; in 2023, the N_2_ level significantly increased yield by 17.61% and 8.64% compared to the N_1_ and N_3_ levels, respectively.

### 2.3. Fruit Quality

#### 2.3.1. Appearance Quality

**Figure 2:**
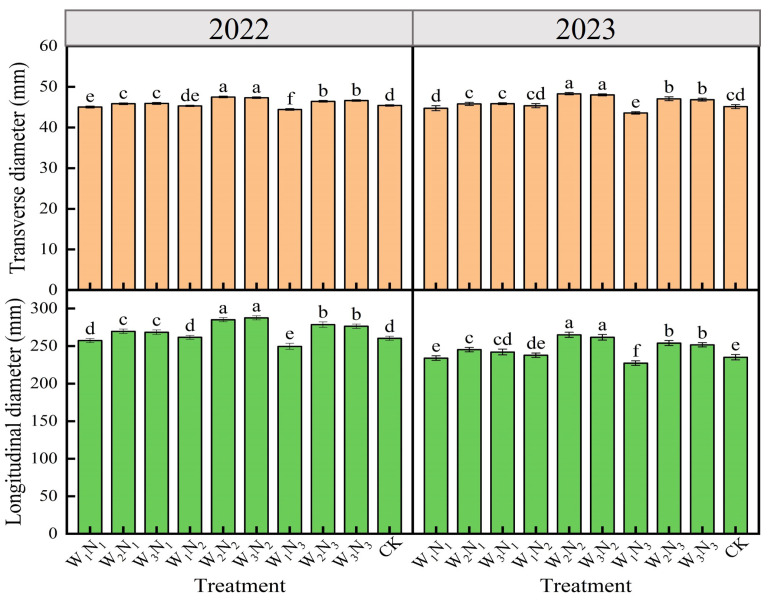
Effects of different water and nitrogen treatments on the appearance quality of eggplant in 2022 and 2023. Different lowercase letters indicate significant differences between treatments at the *p* < 0.05 level. The bars represent the standard deviation.

The changes in eggplant appearance quality under various water and nitrogen treatments in 2022 and 2023 were illustrated in Figure 2. The trends in the variations in fruit transverse and longitudinal diameters were largely consistent across the two years. The transverse diameter ranged from 43.56 to 48.28 mm, and the longitudinal diameter varied between 244.76 and 287.64 mm. The W_2_N_2_ treatment yielded the largest transverse diameter, which was not significantly different (*p* > 0.05) from the W_3_N_2_ treatment but significantly (*p* < 0.05) increased by 4.56% in 2022 and 7.00% in 2023 compared to the CK. The W_1_N_3_ treatment resulted in the smallest longitudinal diameter, which significantly (*p* < 0.05) decreased by 4.27% in 2022 and 3.48% in 2023 compared to the CK. Under the W_2_ and W_3_ levels, both the transverse and longitudinal diameters exhibited a pattern of N_2_ > N_3_ > N_1_, indicating that a moderate water deficit and sufficient irrigation, coupled with a moderate reduction in nitrogen application, were beneficial for enhancing eggplant fruit quality. At the same nitrogen application level, the fruit transverse and longitudinal diameters were the lowest at the W_1_ level, while the longitudinal diameter at the W_2_ level was not significantly different (*p* > 0.05) from that at the W_3_ level. This suggested that eggplant fruit quality initially increased and then decreased with the increase in both irrigation and nitrogen application, and therefore, appropriate water and nitrogen conservation was advantageous for improving eggplant fruit quality.

#### 2.3.2. Nutritional Quality

1. Soluble Protein (SP)

Irrigation, nitrogen application, and their interaction all significantly (*p* < 0.01) affected the SP content (Table 2). The highest SP content was found in the W_2_N_2_ treatment, significantly (*p* < 0.05) increased by 44.76% in 2022 and 33.19% in 2023 compared to the CK, and significantly increased by 5.56% to 67.03% in 2022 and 8.04% to 51.47% in 2023 compared to other water and nitrogen treatments. The lowest SP content was treated with W_1_N_3_ treatment, which significantly decreased by 15.38% in 2022 and 13.73% in 2023 compared to the CK. Under the same irrigation level, SP content first increased and then decreased with the increase in nitrogen application; the highest SP content was observed at the N_2_ level. This indicated that moderate nitrogen reduction was beneficial for increasing the SP content. At the N_1_ level, the W_3_ level was found to have the lowest SP content, with no significant difference (*p* > 0.05) noted between the W_1_ and W_2_ levels. At the N_2_ level, the SP content showed an increasing and then decreasing trend with the increase in irrigation water volume. The W_2_ level significantly increased the SP content by 8.19% compared to the W_1_ level and by 5.56% compared to the W_3_ level in 2022, and by 10.75% and 8.04%, respectively, in 2023. At the N_3_ level, the SP content at the W_3_ level was significantly higher than that at the W_1_ and W_2_ levels. This suggested that moderate water and nitrogen deficiency could help increase the SP content in eggplants.

2. Soluble Sugar Content (SSC)

In 2022, irrigation, nitrogen application, and their interaction all significantly (*p* < 0.01) affected the SSC. In 2023, both irrigation and nitrogen application significantly (*p* < 0.01) affected the SSC, and their interaction had a significant (*p* < 0.05) effect (Table 2). The lowest SSC was observed with the W_1_N_3_ treatment, at 1.98% in 2022 and 2.45% in 2023, with no significant difference (*p* > 0.05) compared to the W_1_N_1_ treatment, while it was significantly lower than the CK by 37.88% in 2022 and 8.57% in 2023. Under the same irrigation level, the SSC mean value was highest at the W_2_ level and lowest at the W_1_ level, indicating that moderate water deficit significantly reduced SSC. Under the same nitrogen application level, the highest mean value of SSC was recorded at the N_2_ level, significantly increased by 31.62% compared to N_1_ and 36.48% compared to N_3_ in 2022, and by 15.47% compared to N_1_ and 14.18% compared to N_3_ in 2023. This suggested that moderate nitrogen reduction was beneficial for SSC accumulation, while excessive nitrogen application could decrease SSC.

3. Soluble Solids (TSS)

As shown in Table 2, nitrogen application and the interaction of water and nitrogen significantly (*p* < 0.01) affected the TSS content over the two-year trial. In 2022, irrigation significantly (*p* < 0.01) impacted TSS content, while in 2023, it had no significant (*p* > 0.05) effect. The TSS content was observed to be at its lowest with the W_1_N_3_ treatment, which resulted in a significant decrease of 9.23% in 2022 and 6.39% in 2023 compared to the CK (*p* < 0.05). The highest TSS content was found in the W_2_N_2_ treatment, which significantly increased by 15.49% in 2022 and 15.47% in 2023 compared to the CK. In terms of the mean TSS content at the same irrigation level, the W_2_ level was observed to have the highest, slightly above the W_3_ level but without significant difference. The TSS content at the W_1_ level was significantly lower than the W_2_ level by 3.43% in 2022 and 2.90% in 2023, indicating that moderate water deficiency significantly reduced TSS, while mild water deficiency had no significant impact on TSS content. In the context of nitrogen application levels, the N_2_ level was noted for its highest mean TSS content, which was significantly elevated compared to the N_1_ and N_3_ levels. This indicated that a moderate reduction in nitrogen fertilizer application was conducive to the accumulation of TSS, thereby enhancing the overall quality of eggplant fruits.

4. Vitamin C (VC)

In 2022, nitrogen application and the interaction of water and nitrogen extremely significantly (*p* < 0.01) affected VC content, and irrigation significantly (*p* < 0.05) impacted VC content. In 2023, irrigation, nitrogen application, and their interaction all highly significantly (*p* < 0.01) affected VC content (Table 2). The range of VC content over the two growing seasons was 41.24 to 69.41 mg·kg^−1^. The W_2_N_2_ treatment had the highest VC content, significantly (*p* < 0.05) increased by 39.53% in 2022 and 50.27% in 2023 compared to the CK. The W_1_N_3_ treatment had the lowest VC content, significantly reduced by 10.16% in 2022 compared to the CK, with no significant difference (*p* > 0.05) in 2023. The mean VC content at the W_2_ level was the highest, significantly higher than the W_1_ and W_3_ levels, which showed no significant difference. This indicated that a mild water deficit was beneficial for increasing VC content. The highest mean VC content was recorded at the N_2_ level, which was significantly higher by 20.16% compared to the N_1_ level and by 30.01% compared to the N_3_ level in 2022, and by 21.91% and 30.79% in 2023, respectively. This suggested that a moderate reduction in nitrogen application was beneficial for increasing the VC content, whereas an excessive application of nitrogen could result in a decrease in VC content.

### 2.4. Water and Nitrogen Use Efficiency

#### 2.4.1. Water Use Efficiency

**Figure 3:**
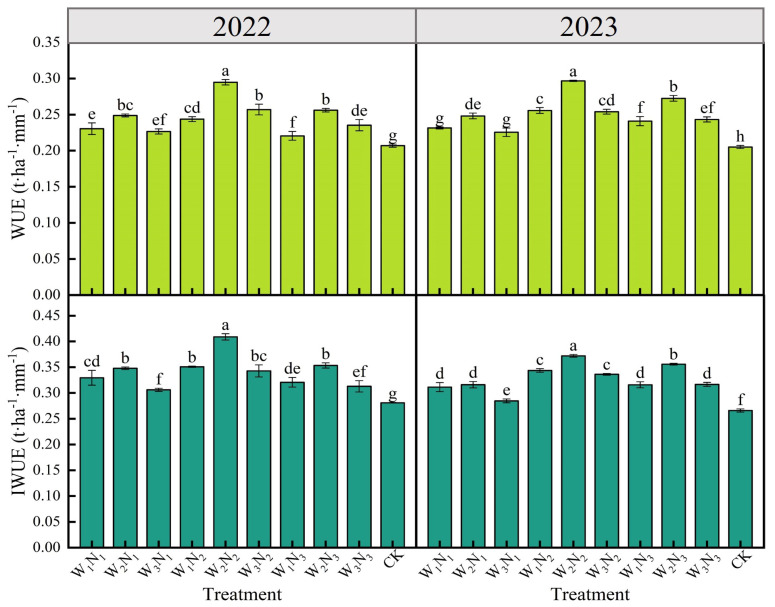
Effects of different water and nitrogen treatments on WUE and IWUE of eggplant in 2022 and 2023. Different lowercase letters indicate significant differences between treatments at the *p* < 0.05 level. The bars represent the standard deviation.

Irrigation, nitrogen application, and their interaction significantly (*p* < 0.01) affected the water use efficiency (WUE) of eggplants (Table 3). The WUE of eggplants varied from 0.205 to 0.297 t·ha^−1^·mm^−1^ across two growing seasons (Figure 3), with the W_2_N_2_ treatment exhibiting the highest WUE, which was significantly (*p* < 0.05) higher by 42.51% (2022) and 44.88% (2023) compared to the CK treatment. At a consistent level of water deficit, WUE initially rose and then declined as nitrogen application increased. At the N_2_ level, WUE peaked, showing a significant improvement of 6.09% to 13.22% compared to the N_1_ level in 2022 and 10.34% to 19.76% in 2023. Compared to the N_3_ level, there was also a significant enhancement of 10.34% to 19.76% in 2022 and 4.53% to 8.79% in 2023. At a uniform nitrogen application rate, the WUE of eggplants showed a trend of increasing and then decreasing as water deficiency intensified, with the W_2_ level achieving the highest WUE. In 2022, W_2_ significantly surpassed W_1_ and W_3_ by 8.26% to 20.90% and 8.94% to 14.79%, respectively. In 2023, W_2_ continued to significantly outperform W_1_ and W_3_, by 6.90% to 16.02% and 9.73% to 16.93%, respectively. This suggested that a moderate water deficit significantly boosts WUE, while both excessive water scarcity and ample water supply could lead to diminished WUE.

#### 2.4.2. Irrigation Water Use Efficiency

Table 3 revealed that irrigation, nitrogen application, and water and nitrogen interactions had a highly significant (*p* < 0.01) impact on the irrigation water use efficiency (IWUE) of eggplants. Over two growing seasons, the IWUE of various water and nitrogen treatments ranged from 0.271 to 0.409 t·ha^−1^·mm^−1^ (Figure 3). The CK treatment exhibited the lowest IWUE, which was significantly (*p* < 0.05) reduced by 8.90% to 45.55% in 2022 and 7.01% to 40.22% in 2023 compared to other treatments. The W_2_N_2_ treatment had the highest IWUE, which was significantly increased by 15.86% to 45.55% in 2022 and 4.68% to 40.22% in 2023 compared to other treatments. In 2023, under identical irrigation conditions, the IWUE of eggplants initially peaked with nitrogen application and then declined. The N_2_ level peaked with IWUE, which was significantly higher by 10.38% to 18.28% compared to N_1_ and by 4.68% to 9.01% compared to N_3_. The 2022 IWUE trend mirrored that of 2023, suggesting that lower nitrogen fertilizer applications substantially improved eggplants’ IWUE. At uniform nitrogen application rates, eggplant IWUE initially rose and then fell with increasing water deficit severity, with the W_2_ level consistently outperforming W_1_ and W_3_ in IWUE. The N_2_ level achieved the highest average IWUE. In 2022, the IWUE at the N_1_ and N_3_ levels were notably 11.89% and 11.55% lower than N_2_, respectively. In 2023, these reductions were even more pronounced, with N_1_ and N_3_ levels being 15.48% and 6.55% lower than N_2_, respectively.

#### 2.4.3. Nitrogen Partial Factor Productivity

**Figure 4:**
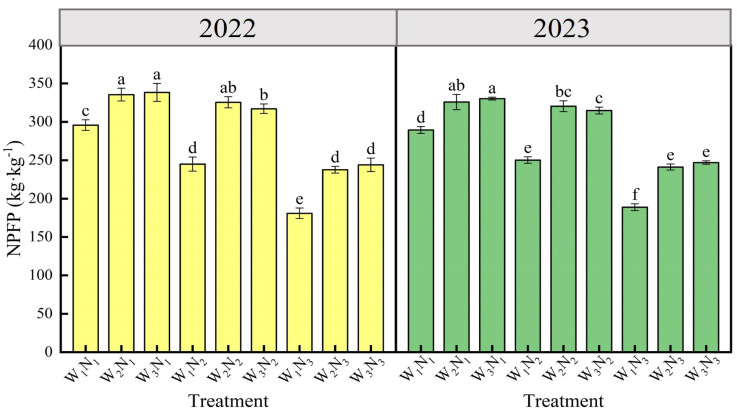
Effects of different water and nitrogen treatments on NPFP of eggplant in 2022 and 2023. Different lowercase letters indicate significant differences between treatments at the *p* < 0.05 level. The bars represent the standard deviation.

Irrigation, nitrogen application, and their interactions significantly (*p* < 0.01) affected the nitrogen partial factorial productivity (NPFP) of eggplants (Table 3). Figure 4 showed that the W_3_N_1_ treatment achieved the highest NPFP, while the W_1_N_3_ treatment had the lowest, with a significant (*p* < 0.05) reduction of 86.98% in 2022 and 74.82% in 2023 for the latter compared to the former. The average NPFP declined with escalating water deficit at a constant irrigation level, with no discernible difference (*p* > 0.05) between the W_2_ and W_3_ levels. However, the W_1_ level markedly underperformed compared to W_2_ and W_3_ by 24.51% and 24.64% in 2022, and 21.80% and 22.43% in 2023, respectively. This suggested that a moderate water deficit could substantially reduce NPFP, whereas a mild deficit did not significantly affect it. Additionally, the average NPFP decreased with increased nitrogen application at a consistent nitrogen level; N_2_ was significantly lower than N_1_ by 9.24% in 2022 and 6.78% in 2023, and N_3_ was significantly lower than N_2_ by 33.93% in 2022 and 30.77% in 2023. This suggested that a reduced nitrogen application could notably enhance the NPFP of eggplants.

### 2.5. Comprehensive Evaluation

#### 2.5.1. Correlation Analysis

**Figure 5:**
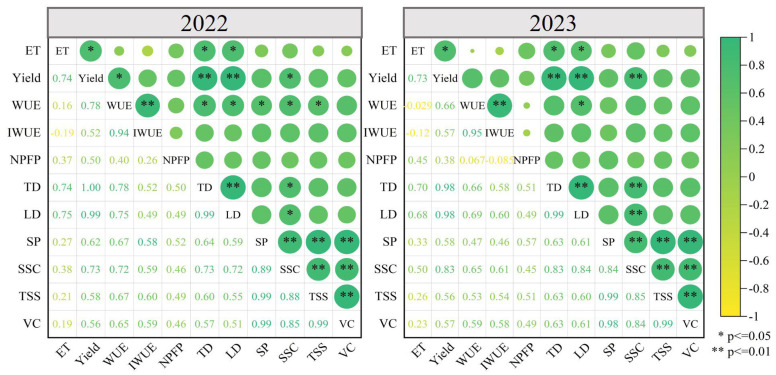
Correlations between water consumption, yield, quality, and water and nitrogen use of eggplant in 2022 and 2023. The gradient of the legend is determined by the strength of correlation, with positive or negative correlation indicated by the color of the circle (positive correlation is indicated in green and negative correlation in yellow). The size of the circle indicates the strength of the correlation, and the number in the box is the correlation coefficient. * and ** indicate significance at the 0.05 and 0.01 levels, respectively. ET, water consumption; WUE, water use efficiency; IWUE, irrigation water use efficiency; NPFP, nitrogen fertilizer bias productivity; TD, transverse diameter of fruits; LD, longitudinal diameter of fruits; SP, soluble protein content; SSC, soluble sugar content; TSS, soluble solids content; VC, vitamin C content.

To ascertain the precision and dependability of the assessment outcomes, a Pearson correlation analysis was performed on the diverse indicators of eggplants, quantifying the linear associations among them. Figure 5 depicts the correlations among water consumption, yield, fruit quality, and water–nitrogen use efficiency for eggplants in 2022 and 2023. Taking the data of 2022 as an example, water consumption was positively (*p* < 0.05) correlated with yield, fruit transverse, and longitudinal diameter. Yield was significantly and positively (*p* < 0.01) correlated with fruit transverse and longitudinal diameter and soluble solids content (SSC). Water use efficiency (WUE) was positively (*p* < 0.05) correlated with fruit transverse diameter and significantly (*p* < 0.01) correlated with irrigation water use efficiency (IWUE). Fruit transverse diameter was significantly and positively (*p* < 0.01) correlated with fruit longitudinal diameter and SSC. SP, SSC, TSS, and VC were all significantly and positively intercorrelated (*p* < 0.01), indicating similar response patterns to water and nitrogen deficiencies.

#### 2.5.2. Optimization of Water and Nitrogen Combination for Eggplant Based on the EWM-TOPSIS Model

The response of eggplant to water and nitrogen regulation under subsurface drip irrigation is characterized by randomness and uncertainty. To minimize potential biases in the comprehensive evaluation caused by highly correlated indicators, Pearson correlation analysis was conducted, leading to the exclusion of indicators such as IWUE, TD, LD, SP, and SSC, while retaining ET, Y, WUE, NPFP, TSS, and VC. By considering the water consumption, yield, water and nitrogen use efficiency, and quality of eggplant comprehensively, the EWM-TOPSIS model was employed for the integrated evaluation, thereby identifying the optimal irrigation and nitrogen application strategy for the Hexi region. Based on the standardized matrix, the entropy weight method was applied to calculate the weights of various indicators for 2022 and 2023 (Table 4). The TOPSIS model was employed to ascertain the distances between various treatments and both the positive and negative ideal solutions, along with the degree of closeness and the ranking of scores (Table 5). The results showed that the evaluation scores for 2022 and 2023 were largely consistent. The W_2_N_2_ treatment achieved the highest score (0.775 in 2022 and 0.841 in 2023), followed by the W_3_N_2_ treatment (0.748 in 2022 and 0.754 in 2023). The W_1_N_3_ treatment had the lowest score (0.064 in 2022 and 0.112 in 2023). The comprehensive analysis of the results from both 2022 and 2023 concluded that implementing a moderate water deficit (60–70% in FC) during the eggplants’ flowering and fruiting stage, paired with a moderate nitrogen application (270 kg·ha^−1^), constituted the most effective irrigation and fertilization strategy for eggplants under sub-mulch drip irrigation in the Hexi region.

## 3. Discussion

### 3.1. Impact of Water-Saving and Nitrogen Reduction on Crop Yields

The results of a two-year experiment demonstrated that irrigation, nitrogen application, and their interaction all significantly (*p* < 0.01) impacted the yield of eggplants. Under the same irrigation level, eggplant yield showed an increasing and then decreasing trend with the increase in nitrogen application. The N_2_ level showed the highest total yield, which indicated that appropriate nitrogen reduction could effectively enhance crop yield. The appropriate amount of nitrogen fertilizer can promote leaf growth, improve crop photosynthesis, accelerate the synthesis of photosynthetic products, and thereby increase nutrient accumulation and yield [18]. Under the same nitrogen application level, there was no significant difference (*p* > 0.05) in yield between the W_2_ and W_3_ levels, while the yield at the W_1_ level was significantly (*p* < 0.05) reduced by 13.45–32.83% (2022) and 12.57–28.03% (2023) compared to the W_2_ level. This indicated that a mild water deficit did not decrease eggplant yield, whereas a moderate water deficit significantly inhibited it. This may be due to crops adopting strategies such as slowing down growth rates and reducing transpiration when under mild water stress to maintain water balance [19], prioritizing fruit development, and thus not significantly affecting eggplant yield. Under moderate water stress conditions, crops may experience growth stagnation, reduced nutrient absorption, and hindered photosynthesis [20]. At such times, eggplant plants prioritized the supply of water to growth points and leaves, leading to a decrease in eggplant yield. The W_2_N_2_ treatment showed the highest eggplant yield (87.87 t·ha^−1^ in 2022 and 86.50 t·ha^−1^ in 2023), with no significant difference (*p* > 0.05) compared to the W_3_N_2_ treatment, and significantly (*p* < 0.05) increased by 35.06% (2021) and 36.91% (2023) compared to the CK, consistent with previous research findings [21]. A scientifically sound ratio of water to nitrogen can promote the plant’s absorption, transformation, and utilization of water and nutrients; promote plant growth; improve photosynthetic efficiency; and coordinate the accumulation and distribution of photosynthetic products, which is key to achieving high crop yields.

### 3.2. Impact of Water-Saving and Nitrogen Reduction on Fruit Quality

Proper water and nitrogen management are key to enhancing fruit quality. Improper irrigation and nitrogen application, such as excessive watering or over-fertilization, can adversely affect fruit quality. Under uniform irrigation conditions, the transverse and longitudinal diameters of eggplant fruit demonstrated a notable increase with the initial increment of nitrogen application, yet these dimensions were observed to taper off after a certain nitrogen threshold was exceeded. Notably, it was at the N_2_ nitrogen level that the fruit achieved their maximum girth in both transverse and longitudinal measurements. Nitrogen deficiency was found to result in foliar chlorosis and stunted growth, significantly impairing the plant’s ability to assimilate and transport nutrients [22]. Conversely, an excess of nitrogen has been identified to promote excessive vegetative growth and lead to an inequitable allocation of nutrients, both scenarios acting to restrict the enhancement of the fruit’s transverse and longitudinal diameters. Under the same nitrogen application levels, the transverse and longitudinal diameters of the fruit demonstrated an initial increase followed by a decrease as the severity of the water deficit intensified. There was no significant difference (*p* > 0.05) in the horizontal and vertical diameters of fruits between the W_2_ and W_3_ levels, while the W_1_ level significantly (*p* < 0.05) reduced them, indicating that mild water deficit did not affect the transverse and longitudinal diameters of eggplant fruit. This may be because, under mild water stress, plants can regulate stomatal opening to reduce water transpiration, maintaining water balance [23], and allowing for normal growth and development without affecting the fruit. However, moderate water stress has been observed to diminish the crop’s capacity for the absorption and utilization of water and nutrients, and it can also disrupt cellular elongation and division processes [24], thereby significantly decreasing the fruit’s transverse and longitudinal diameters. This indicated that cellular processes were preserved under mild conditions but were compromised by moderate stress, affecting fruit dimensions.

The findings of this study revealed that, under constant irrigation levels, the SP, SSC, TSS, and VC in eggplant fruits exhibited a trend of initial increase followed by a decrease with escalating nitrogen application, reaching their zenith at the N_2_ level. Moderate increases in nitrogen fertilizer application have been shown to enhance the SSC, TSS, OA, and VC content in greenhouse cherry tomatoes, whereas excessive nitrogen application may result in a decline in these quality indicators [25]. This suggested that an optimized nitrogen application rate was crucial for maximizing fruit quality in eggplant cultivation. This was because an appropriate amount of nitrogen fertilizer was beneficial for promoting protein synthesis and maintaining metabolic balance within the plant, thereby improving crop quality, whereas an overabundance of nitrogen could lead to metabolic disorders in protein synthesis, an imbalance in the plant’s carbon-to-nitrogen ratio, and a disruption in material metabolism, which negatively impacted the quality of eggplant fruit.

Under the W2 water level, the eggplant fruits demonstrated the highest average content of SP, SSC, TSS, and VC, suggesting that mild water stress during the flowering and fruiting stage was beneficial for enhancing the nutritional quality of eggplant fruits. Mild water stress led to an imbalance in cellular osmotic pressure [26], at which time the plant actively synthesized and accumulated soluble substances to maintain stable osmotic pressure, resulting in increased levels of soluble protein and soluble sugars. Additionally, mild water stress-induced oxidative stress in plants [27], which promoted the synthesis of antioxidant substances within cells, such as VC, and affected the synthesis and transport of growth regulators [28], facilitating the accumulation of soluble solids. The optimal quality indicators under the moderate water and nitrogen (W2N2) treatment demonstrated that proper water and nitrogen regulation could significantly improve the quality of eggplant fruits, consistent with the findings of Yue [29] et al. on tomatoes, where the best content of vitamin C, soluble protein, and lycopene was achieved with an irrigation volume of 1.0 ETc and a nitrogen application rate of 320 kg·ha^−1^. Liu [30] and colleagues found that an application rate of nitrogen between 80 and 140 kg·ha^−1^, with an irrigation volume of 5000–8000 m^3^·hm^−2^, could significantly enhance rice yield and quality, while excessive water and fertilizer input may have had the opposite effect.

### 3.3. Impact of Water-Saving and Nitrogen Reduction on WUE, IWUE and NPFP

In scenarios of water scarcity and excessive fertilizer use, improving the efficiency of water and nitrogen use efficiencies is key to achieving sustainable agriculture. The effects of irrigation, nitrogen application, and their interactions on eggplant’s WUE, IWUE, and NPFP were all highly significant (*p* < 0.01). Under stable irrigation and nitrogen application conditions, both the WUE and IWUE of eggplants exhibited a pattern of initial increase and subsequent decline as nitrogen and water inputs were escalated. The combination of moderate water deficit and moderate nitrogen application (W_2_N_2_) yielded the highest WUE and IWUE, which were significantly (*p* < 0.05) increased by 14.79% to 42.51% in 2022 and 8.79% to 44.88% in 2023 for WUE, and by 15.86% to 45.55% in 2022 and 4.68% to 40.22% in 2023 for IWUE, compared to other water–nitrogen treatments. Mild water stress enhanced water use efficiency by stimulating root development and optimizing stomatal function [31]. Moderate nitrogen application promoted the absorption and transport of water, leading to improved crop nutrition. The synergistic effect of these two factors led to higher efficiency in the use of water and irrigation, allowing for water and fertilizer conservation while maintaining high crop yield and quality. Studies by Hao et al. [32] indicated that apple yield and overall water use efficiency followed an ascending then descending trend with increased water and nitrogen input, with the optimal irrigation and nitrogen application for mountain apple trees being an interval of 95–115 mm and a rate of 470–575 kg·ha^−1^, respectively. Yang et al. [33] concluded that under subsurface drip irrigation, the highest yield and water use efficiency for potatoes in arid northwest regions were achieved with a soil moisture ratio of 40–50% and a nitrogen application rate of 135–150 kg·ha^−1^.

Nitrogen partial factorial productivity (NPFP) is a crucial indicator for measuring the efficiency of nitrogen fertilizer use in agriculture, playing a significant role in improving agricultural production efficiency and reducing costs. Under the same level of water deficit, eggplant NPFP decreased with increased nitrogen application; under the same nitrogen application level, mild water deficit had no significant (*p* > 0.05) effect on NPFP, while moderate water deficit significantly (*p* < 0.05) reduced NPFP. This indicated that reducing nitrogen fertilizer use and increasing irrigation water could significantly (*p* < 0.05) enhance eggplant NPFP. Wang et al. [21] obtained similar results, showing that greenhouse sweet pepper NPFP decreased with increased nitrogen application, and reducing nitrogen fertilizer application significantly improved NPFP. Both excessive water deficit and over-application of nitrogen can negatively impact eggplant NPFP. This may be because over-application of nitrogen increases soil nitrogen concentration, leading to an internal nutrient imbalance in plants [34], and consequently a decrease in NPFP. Additionally, moderate water deficit could also reduce plant water use efficiency, further affecting nitrogen partial factorial productivity. The results of Li et al. [35] study on winter wheat in the North China Plain region are also consistent with the results of this experiment.

### 3.4. Determination of the Optimal Irrigation Nitrogen Application Management Model

Different water–nitrogen treatments had varying impacts on various indices of eggplants, making it challenging to identify an irrigation and nitrogen application regime that optimized all indices. Therefore, considering the water consumption, yield, water–nitrogen use efficiency, and quality of eggplants, a scientific and comprehensive evaluation of each water–nitrogen treatment was conducted using the EWM-TOPSIS method. The results showed that in two years of trials, the W_2_N_2_ treatment received the highest score (0.775 in 2022 and 0.841 in 2023). Considering all factors, a mild water deficit during the flowering and fruiting period (60–70% in FC) combined with moderate nitrogen application (270 kg·ha^−1^) was identified as the optimal irrigation and nitrogen management model for subsurface drip-irrigated eggplants in the study area.

## 4. Materials and Methods

### 4.1. Overview of the Experimental Area

**Figure 6:**
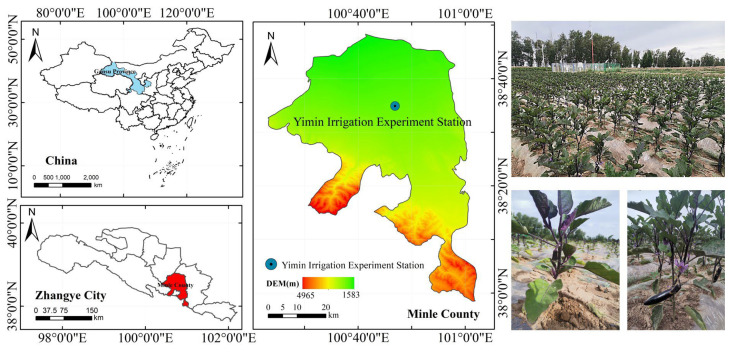
Geographical location of the experimental area (Minle County, China).

The experiments were conducted in 2022 and 2023 at the Yimin Irrigation Experiment Station in Minle County, Gansu Province of northwest China (100°47′ E, 38°35′ N, and approximately 1970 m a.s.l.) (Figure 6). This region had a continental desert steppe climate, with abundant sunlight and a dry atmosphere. The average annual temperature is about 7.6 °C, and the annual precipitation ranges from 183 to 285 mm, mainly concentrated in June, July, and August. The evaporation rate is as high as 1638 mm, with approximately 3000 h of sunshine per year, and the frost-free period lasts from 109 to 174 days. The experimental area’s soil is a loamy soil with a flat structure and moderate fertility. The soil bulk density within the 0~60 cm layer is approximately 1.48 g·cm^−3,^ and the soil’s field capacity is 24.0%. The soil contains 11.3 g·kg^−1^ of organic matter, 22.67 mg·kg^−1^ of available phosphorus, and 104.32 mg·kg^−1^ of available potassium. Additionally, the groundwater level is deeper than 20 m, indicating that the area is not affected by salinization issues.

### 4.2. Experimental Design

The experiment utilized a randomized complete block design as shown in Table 6. Soil moisture was categorized into three gradients: moderate water deficit (W_1_, soil water content maintained at 50–60% of field capacity, FC), mild water deficit (W_2_, soil water content maintained at 60–70% of FC), and full irrigation (W_3_, with soil water content maintained at 70–80% of FC). Nitrogen fertilizer application was also set at three levels: low nitrogen (N_1_, 215 kg·ha^−1^), medium nitrogen (N_2_, 270 kg·ha^−1^), and high nitrogen (N_3_, 325 kg·ha^−1^). Mild and moderate water deficits were applied during eggplant flowering and fruiting, while full irrigation was supplied during other growth stages. The control class CK received full irrigation (W_3_) throughout the entire growth period without nitrogen application (N_0_). Therefore, the experiment consisted of 9 different water–nitrogen management treatments and one control, with three replications in each treatment and control, totaling 30 experimental plots with a plot size of 8 m^2^ (2 m × 4 m). The detailed experimental design was seen in Table 6. Soil moisture was regulated in the 0–60 cm soil layer and measured every 8 days for each treatment. Irrigation was required when soil moisture content approached the lower design irrigation limit to bring it up to the upper limit.

The experimental eggplant variety was selected as “Lanza No. 2”, which has robust growth, thick stems and large leaves, and is resistant to epidemics and virus diseases. The growth stages of the eggplant were delineated into the seedling period, flowering and fruiting period, full fruiting period, and later fruiting period (Table 7). Prior to transplanting, the experimental plots were prepared by mechanical plowing to a depth of 30 cm for soil tillage and leveling, followed by mechanical weeding. Manual furrows were created one to two days before transplanting to apply the base fertilizer. Equal rates of calcium superphosphate (P_2_O_5_ ≥ 12%, 200 kg·ha^−1^) and potassium sulfate (K_2_O ≥ 51%, 350 kg·ha^−1^) were used, along with 40% of the total urea (total *n* ≥ 46.4%) as part of the base dressing. The remaining 60% of urea was top-dressed in increments of 20% during the flowering and fruiting period, the full fruiting period, and the later fruiting period, respectively, using a simple venturi injector for fertigation.

**Figure 7:**
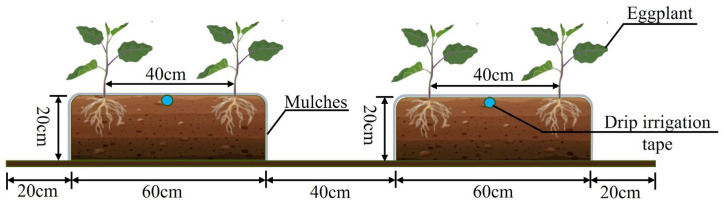
Schematic diagram of field layout of eggplant.

Eggplant seedlings were transplanted on 11 May 2022 and 14 May 2023. An open-field cultivation model with two ridges per plot, each ridge accommodating two rows, was utilized (Figure 7). The ridges were 60 cm wide, 20 cm high, with a 40 cm space between them. A drip irrigation belt with a drip head spacing of 30 cm and a drip head flow rate of 2.4 L·h^−1^ was laid in the middle of each row and covered with a layer of colorless plastic film with a width of 120 cm. The seedlings were transplanted on both sides of the ridges with a row spacing of 40 cm and a plant spacing of 40 cm. To prevent water seepage between adjacent plots, an impermeable membrane was installed 0.6 m deep in each plot. Irrigation volumes were controlled using a water meter with an accuracy of 0.0001 m^3^. Throughout the two-year study, routine field management, including pruning, weeding, and pest control, was consistently performed across all plots. The eggplant fruits were harvested in four batches on the following dates: 15 July, 31 July, 14 August, and 30 August in 2022, and 15 July, 29 July, 14 August, and 31 August in 2023.

### 4.3. Measurements and Calculations

#### 4.3.1. Meteorological Information

**Figure 8:**
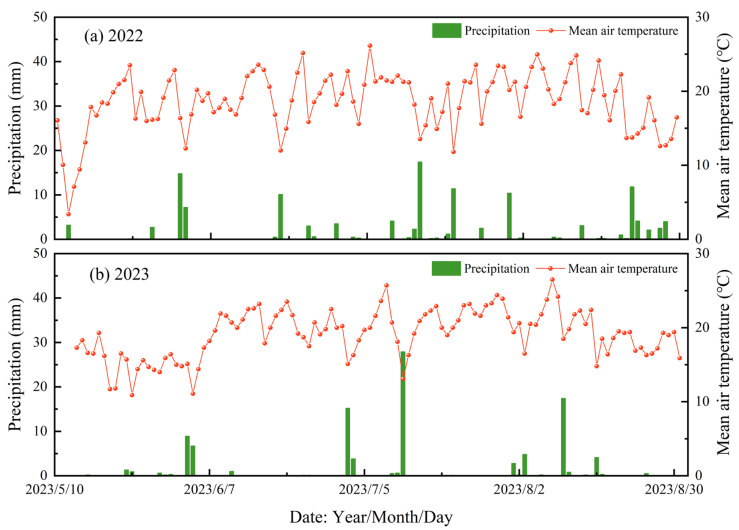
Changes in precipitation and mean air temperature during May–August in the study area in 2022 (**a**) and 2023 (**b**).

An automatic meteorological station of the Zhonghuan Tianyi DZZ6 model was utilized to monitor meteorological parameters such as air temperature, precipitation, and sunshine duration, with an automated system employed for data logging. The average air temperature and precipitation for the years 2022 and 2023 are depicted in Figure 8.

#### 4.3.2. Soil Moisture Content

Soil moisture content was determined using the oven-drying method. A preliminary measurement of soil moisture was taken before eggplant transplanting, followed by measurements every 8 days, with additional measurements after rainfall or irrigation. Within each experimental plot, soil samples were collected at five different soil depth intervals (0–20 cm, 20–40 cm, 40–60 cm, 60–80 cm, 80–100 cm) using a soil auger at the midpoint of a line connecting two adjacent plants chosen randomly on one side, with a total soil sampling depth of 100 cm. Considering that the 0–50 cm depth was the main active region of the eggplant root system, the average soil moisture content within the 0–60 cm soil layer was used as the reference baseline for soil moisture content in the planned moist layer.

The soil water content and irrigation volume were calculated as follows [36]:

(1) $$\omega_{i}=\frac{m_{ia}-m_{ib}}{m_{ib}}\times 100\%,$$

(2) $$M=10\gamma HP(\omega_{j}-\omega_{i}),$$

where *ω_*i*_* represents the mass water content of the *i*th soil layer (%); *m_ia_* is the weight of the fresh soil in the *i*th layer (g); *m_ib_* is the weight of the dry soil in the *i*th layer (g); *M* is the irrigation volume (mm); *γ* is the soil bulk density of the planned wetted layer (g·cm^−3^); *H* is the depth of the planned wetted layer (cm); *P* is the drip irrigation design wetting ratio (%); *ω_j_* is the upper limit of soil moisture content in the experimental design (%); *ω_i_* is the actual measured soil moisture content (%).

#### 4.3.3. Water Consumption

The water balance equation is utilized to estimate the stage water consumption of eggplants [37].

(3) $$ET=10\sum_{i=1}^{n}\gamma_{i}H_{i}(\omega_{i1}-\omega_{i2})+M+P+K-C,$$

where *ET* represents water consumption of eggplants at the plant growth stage (mm); *i* is the order of soil layer; *n* is 3 (calculation layer every 20 cm); *γ_i_* is the soil bulk density of the *i*th layer; *H_i_* is the thickness of the *i*th soil layer (cm); *ω_i_*_1_ and *ω_i_*_2_ are the soil moisture contents at the beginning and end of the measurement period for the *i*th layer (%); *M* is the irrigation volume during that period (mm); *P* is the effective rainfall during the period (mm); *K* is the groundwater recharge during the period (mm); *C* is the deep percolation volume during the period. At the experimental site, the groundwater table is deeper than 20 m, making it inaccessible for crop uptake; hence, *K* = 0. Since the experimental field is relatively flat and the irrigation method is drip irrigation, the designed irrigation volume is lower than the soil moisture content, preventing deep percolation, thus *C* = 0.

#### 4.3.4. Yield

In each plot, three uniform eggplants were selected and tagged for yield measurement, which was used to serve as the subjects for the next yield assessment. The yield in each plot was calculated based on the sum of all the individual plant yields, and the average yield in the three replicate plots was used as the yield in each treatment [8].

#### 4.3.5. Water Use Efficiency, Irrigation Water Use Efficiency, and Nitrogen Partial Factor Productivity

In each plot, three uniform eggplants were selected and tagged for yield measurement, which was used to serve as the subjects for the next yield assessment. The yield in each plot was calculated based on the sum of all the individual plant yields, and the average yield in the three replicate plots was used as the yield in each treatment.

Water use efficiency (WUE), irrigation water use efficiency (IWUE), and nitrogen partial factor productivity (NPFP) are calculated as follows:

(4) WUE=Y/ET,

(5) IWUE=Y/I,

(6) $$NPFP=Y_{N}/F_{N},$$

where *WUE* is water use efficiency (t·ha^−1^·mm^−1^); *IWUE* is the irrigation water use efficiency (t·ha^−1^·mm^−1^); *NPFP* is nitrogen partial factor productivity (kg·kg^−1^). *Y* is the eggplant yield per unit area (t·ha^−1^); *ET* is water consumption of eggplant (mm); *I* is the irrigation amount of eggplant during the whole growth period (mm); *F_N_* is the amount of nitrogen fertilizer applied (kg·ha^−1^).

#### 4.3.6. Fruit Quality

1. Appearance quality

Eggplant fruit diameters were measured and recorded using a digital vernier caliper with an accuracy of 0.01 mm. The transverse diameter was measured three times at different locations on the fruit and averaged, as was the longitudinal diameter.

2. Nutritional quality

In each plot, five eggplant fruits with bright color, uniform size, and undamaged skin were selected. Rinse with distilled water and dry the epidermis with filter paper, remove the purple epidermis of eggplant, weigh the middle part of the fruit as required for grinding and mixing, and determine the nutritional quality of eggplant fruits. The soluble protein (SP) content was determined using the Coomassie Brilliant Blue G-250 dye-binding method [38]; the soluble sugar (SSC) content was measured using the anthrone colorimetric method [39]; the soluble solids (TSS) content was assessed with a WAY-2S type Abbe refractometer; and vitamin C was quantified using the molybdenum blue colorimetric method [40].

#### 4.3.7. EWM-TOPSIS Models

1. Construction of the comprehensive evaluation index matrix and perform standardization processing [41].

Taking into account eggplant water consumption, yield, quality, and water–nitrogen use efficiency, an evaluation object and index data matrix X was established. Since the control treatment CK does not apply nitrogen, there are a total of 9 evaluation objects and 11 evaluation indicators (water consumption, yield, WUE, IWUE, NPFP, fruit transverse diameter, longitudinal diameter, SP, SSC, TSS, and VC). The original matrix was standardized with the method of polar deviation processing to eliminate the effects of magnitude and order of magnitude.

(7) $$X={\left(x_{ij}\right)}_{m\times n}={\left[\begin{array}{cccc} x_{11} & x_{12} & \cdots & x_{1n}\\ x_{21} & x_{22} & \cdots & x_{2n}\\ \vdots & \vdots & \ddots & \vdots \\ x_{m1} & x_{m2} & \cdots & x_{mn} \end{array}\right]}_{m\times n},$$

(8) $${z_{ij}}^{+}=\frac{x_{ij}-\min_{i}\{x_{ij}\}}{\max_{i}\{x_{ij}\}-\min_{i}\{x_{ij}\}},$$

(9) $${z_{ij}}^{-}=\frac{\max_{i}\{x_{ij}\}-x_{ij}}{\max_{i}\{x_{ij}\}-\min_{i}\{x_{ij}\}},$$

where *x*_*ij*_ represents the *j*th evaluation criterion for the *i*th evaluation object, where *m* = 9 and *n* = 12.

2. Calculation of the *j*th indicator weight for the *i*th evaluation object and construction of a new matrix P.

(10) $$P_{ij}=\frac{z_{ij}}{\sum_{i=1}^{m}z_{ij}}.$$

(11) $$P={\left(P_{ij}\right)}_{m\times n}.$$

3. Calculation of the information entropy value of the jth indicator *e_j_*.

(12) $$e_{j}=-\frac{1}{\ln m}\sum_{i=1}^{m}P_{ij}\ln P_{ij},(j=1,2,\cdots n).$$

(13) $$w_{ej}=\frac{1-e_{j}}{\sum_{j=1}^{n}\left(1-e_{j}\right)}.$$

Multiplication of each standardized matrix element based on the corresponding weight in obtaining the weighted normalized decision matrix.

(14) $$Z=\left[\begin{array}{cccc} z_{11} & z_{12} & \cdots & z_{1n}\\ z_{21} & z_{22} & \cdots & z_{2n}\\ \vdots & \vdots & & \vdots \\ z_{m1} & z_{m2} & \cdots & z_{mn} \end{array}\right]=\left[\begin{array}{cccc} r_{11}w_{1} & r_{12}w_{1} & \cdots & r_{1n}w_{1}\\ r_{21}w_{2} & r_{22}w_{2} & \cdots & r_{2n}w_{2}\\ \vdots & \vdots & & \vdots \\ r_{m1}w_{m} & r_{m2}w_{m} & \cdots & r_{mn}w_{m} \end{array}\right].$$

4. Identification of the positive ideal solution *Z^+^* and the negative ideal solution *Z^−^*.

(15) $$\begin{array}{l} Z^{+}=\left\{\max z_{ij}|j=1,2,\cdots ,n\right\}=\left\{z_{1}^{+},z_{2}^{+},\cdots ,z_{n}^{+}\right\}\\ Z^{-}=\left\{\min z_{ij}|j=1,2,\cdots ,n\right\}=\left\{z_{1}^{-},z_{2}^{-},\cdots ,z_{n}^{-}\right\} \end{array}.$$

5. Calculation of the distance of each evaluation object from the positive and negative ideal solutions, denoted as *d*_*i*_^+^ and *d*_*i*_^−^.

(16) $$\begin{array}{l} d_{i}^{+}=\sqrt{\sum_{j=1}^{n}{\left(z_{ij}-z_{j}^{+}\right)}^{2}},(i=1,2,\cdots ,m)\\ d_{i}^{-}=\sqrt{\sum_{j=1}^{n}{\left(z_{ij}-z_{\bar{j}}\right)}^{2}},(i=1,2,\cdots ,m) \end{array}.$$

6. Computation of the closeness degree of each evaluation object to the optimal solution *C_i_*.

(17) $$T_{i}=\frac{d_{i}^{-}}{d_{i}^{-}+d_{i}^{+}},1\leq i\leq m,$$

where the larger the value of *C*_*i*_, the closer the *i*th evaluation object is to the optimal level. The value of *T_i_* ranges from 0 to 1, where a higher *T_i_* closer to 1 indicates a higher comprehensive score of the evaluation object.

### 4.4. Statistical Analysis

Preliminary calculations and data processing of the experimental data were conducted using Microsoft Excel 2019. Variance analysis (ANOVA) and Pearson correlation analysis were performed on data including water consumption, yield, quality, and water–nitrogen use efficiency using IBM SPSS Statistics 26.0 software. Data processing and EWM-TOPSIS computations were carried out using Matlab 2017b. Origin 2021 software was used for graphical plotting.

## 5. Conclusions

Mild water deficit significantly reduced the water consumption of eggplants but significantly increased yield, water use efficiency (WUE), irrigation water use efficiency (IWUE), fruit diameters (both transverse and longitudinal), and vitamin C content. Moderate nitrogen application significantly enhanced eggplant yield, WUE, IWUE, and fruit quality and reduced the nitrogen partial factorial productivity (NPFP). By taking into account water consumption, yield, water–nitrogen use efficiency, and quality, the EWM-TOPSIS method provided a scientific and comprehensive evaluation of different water–nitrogen treatments. It indicated that applying a mild water deficit (60–70% in FC) during the flowering and fruiting period, with an adequate water supply (70–80% in FC) during the rest of the growth period, and moderate nitrogen application (270 kg·ha^−1^) throughout the growth period, was the optimal irrigation and nitrogen application pattern for this region. The results of this study provide a theoretical basis for water-saving, high-yield, and high-quality cultivation of eggplants in the Hexi Oasis irrigation area of northwest China under cold and arid conditions. However, different degrees of water deficit treatment during various growth stages impact eggplants differently. Therefore, further research is needed on the effects of setting different water deficit levels during each growth stage or continuous deficit irrigation during multiple growth stages on eggplant yield, quality, and water–nitrogen utilization efficiency.

## Figures and Tables

**Table 1:** Effects of different water and nitrogen treatments on total water consumption and yield of eggplant in 2022 and 2023.

Treatment	2022		2023	
	Total Water Consumption (mm)	Yield (t·ha^−1^)	Total Water Consumption (mm)	Yield (t·ha^−1^)
W_1_N_1_	275.88 ± 6.80 e	63.57 ± 1.49 d	268.78 ± 4.68 d	62.22 ± 0.96 e
W_2_N_1_	289.92 ± 5.60 d	72.12 ± 1.80 c	282.24 ± 4.27 c	70.04 ± 2.13 c
W_3_N_1_	321.02 ± 6.35 b	72.74 ± 2.52 c	314.97 ± 10.14 b	70.99 ± 0.40 c
W_1_N_2_	271.40 ± 6.45 e	66.15 ± 2.48 d	264.24 ± 6.61 d	67.56 ± 1.15 d
W_2_N_2_	298.07 ± 6.06 cd	87.87 ± 1.97 a	291.54 ± 6.83 c	86.50 ± 1.90 a
W_3_N_2_	333.13 ± 6.94 a	85.60 ± 1.67 a	334.62 ± 8.96 a	84.97 ± 1.20 a
W_1_N_3_	266.73 ± 7.07 e	58.81 ± 2.25 e	254.90 ± 9.60 d	61.38 ± 1.42 e
W_2_N_3_	301.60 ± 6.41 c	77.23 ± 1.44 b	287.71 ± 8.79 c	78.38 ± 1.27 b
W_3_N_3_	337.19 ± 6.08 a	79.32 ± 2.87 b	330.07 ± 7.61 a	80.28 ± 0.73 b
CK	314.44 ± 6.07 b	65.06 ± 1.92 d	307.99 ± 8.75 b	63.18 ± 1.98 e
Significant lever (F value)				
F_W_	193.48 **	182.79 **	151.51 **	328.34 **
F_N_	6.35 **	75.43 **	5.90 **	197.40 **
F_W×N_	3.44 *	10.44 **	3.03 *	17.07 **

Note: Values are the standard error of the average ± average. Different lowercase letters within a column indicate a significant difference (*p* < 0.05). F_W_: the value of the split-plot ANOVA for water deficit degree; F_N_: the value of the split-plot ANOVA for nitrogen application rate; F_W×N_: the value of the split-plot ANOVA for the interaction between water deficit degree and nitrogen application rate. * *p* < 0.05, ** *p* < 0.01 (Duncan’s multiple range test).

**Table 2:** Effects of different water and nitrogen treatments on nutrient quality of eggplant in 2022 and 2023.

Treatment	2022				2023			
	SP (mg·g^−1^)	SSC (%)	TSS (%)	Vc (mg·kg^−1^)	SP (mg·g^−1^)	SSC (%)	TSS (%)	Vc (mg·kg^−1^)
W_1_N_1_	2.42 ± 0.12 d	2.10 ± 0.10 e	4.44 ± 0.11 d	52.64 ± 1.62 c	2.56 ± 0.10 d	2.47 ± 0.15 e	4.53 ± 0.06 b	53.93 ± 1.79 c
W_2_N_1_	2.51 ± 0.09 cd	2.97 ± 0.11 b	4.51 ± 0.08 d	51.62 ± 1.88 c	2.59 ± 0.11 d	2.88 ± 0.11 b	4.57 ± 0.12 b	55.12 ± 2.73 c
W_3_N_1_	2.19 ± 0.09 e	2.52 ± 0.09 d	4.19 ± 0.08 e	47.38 ± 1.04 d	2.35 ± 0.10 e	2.60 ± 0.11 cd	4.30 ± 0.10 c	48.57 ± 1.79 d
W_1_N_2_	2.81 ± 0.09 b	3.25 ± 0.11 a	4.78 ± 0.08 ab	60.25 ± 1.94 b	2.79 ± 0.13 bc	2.91 ± 0.10 b	4.83 ± 0.12 a	62.26 ± 2.72 b
W_2_N_2_	3.04 ± 0.07 a	3.41 ± 0.09 a	4.92 ± 0.12 a	63.39 ± 1.04 a	3.09 ± 0.12 a	3.15 ± 0.13 a	5.00 ± 0.17 a	69.41 ± 2.73 a
W_3_N_2_	2.88 ± 0.10 b	3.34 ± 0.10 a	4.72 ± 0.10 bc	58.57 ± 2.07 b	2.86 ± 0.12 b	3.13 ± 0.10 a	4.80 ± 0.10 a	60.48 ± 2.06 b
W_1_N_3_	1.82 ± 0.09 g	1.98 ± 0.09 e	3.90 ± 0.10 f	41.24 ± 2.13 e	2.04 ± 0.12 f	2.45 ± 0.17 e	4.07 ± 0.12 d	43.21 ± 1.79 e
W_2_N_3_	2.03 ± 0.09 f	2.47 ± 0.07 d	4.13 ± 0.10 e	44.41 ± 1.76 d	2.17 ± 0.09 ef	2.70 ± 0.15 bc	4.27 ± 0.12 c	47.38 ± 2.06 d
W_3_N_3_	2.59 ± 0.10 bc	2.86 ± 0.11 bc	4.56 ± 0.11 cd	54.50 ± 2.06 c	2.66 ± 0.12 cd	2.89 ± 0.09 b	4.60 ± 0.10 b	56.31 ± 2.06 c
CK	2.10 ± 0.09 ef	2.73 ± 0.08 c	4.26 ± 0.13 e	45.43 ± 1.15 d	2.32 ± 0.09 e	2.66 ± 0.07 cd	4.33 ± 0.12 c	46.19 ± 2.06 de
Significant lever (F value)								
F_W_	13.00 **	80.74 **	5.50 *	3.69 *	5.98 **	16.76 **	3.19 ns	8.00 **
F_N_	128.88 **	168.29 **	67.00 **	116.47 **	54.72 **	24.00 **	42.70 **	88.52 **
F_W×N_	28.91 **	25.93 **	20.85 **	27.81 **	15.01 **	3.02 *	10.54 **	20.32 **

Note: Values are the standard error of the average ± average. Different lowercase letters within a column indicate a significant difference (*p* < 0.05). F_W_: the value of the split-plot ANOVA for water deficit degree; F_N_: the value of the split-plot ANOVA for nitrogen application rate; F_W×N_: the value of the split-plot ANOVA for the interaction between water deficit degree and nitrogen application rate. * *p* < 0.05, ** *p* < 0.01, ns *p* > 0.05 (Duncan’s multiple range test).

**Table 3:** Effects of different water and nitrogen treatments on WUE, IWUE, and NPFP of eggplant in 2022 and 2023.

Treatment	2022			2023		
	WUE (t·ha^−1^·mm^−1^)	IWUE (t·ha^−1^·mm^−1^)	NPFP (kg·kg^−1^)	WUE (t·ha^−1^·mm^−1^)	IWUE (t·ha^−1^·mm^−1^)	NPFP (kg·kg^−1^)
W_1_	0.232 b	0.334 b	240.55 b	0.243 b	0.330 b	242.83 a
W_2_	0.267 a	0.370 a	299.51 a	0.272 a	0.355 a	295.76 b
W_3_	0.231 b	0.311 c	299.81 a	0.232 c	0.307 c	297.29 b
N_0_	0.207 c	0.281 c	—	0.205 d	0.271 d	—
N_1_	0.235 b	0.328 b	323.16 a	0.235 c	0.310 c	315.11 a
N_2_	0.265 a	0.367 a	295.83 a	0.269 a	0.358 a	295.10 b
N_3_	0.237 b	0.329 b	220.88 c	0.252 b	0.336 b	225.67 c
Significant lever (F value)						
F_W_	112.98 **	93.87 **	163.87 **	183.20 **	139.78 **	314.36 **
F_N_	92.85 **	67.24 **	394.47 **	173.52 **	230.48 **	720.17 **
F_W×N_	8.77 **	5.49 **	5.50 **	8.88 **	12.74 **	8.67 **

Note: W and N are the water deficit degree and nitrogen application rate, respectively. Values are the standard error of the average ± average. Different lowercase letters within a column indicate a significant difference (*p* < 0.05). F_W_: the value of the split-plot ANOVA for water deficit degree; F_N_: the value of the split-plot ANOVA for nitrogen application rate; F_W×N_: the value of the split-plot ANOVA for the interaction between water deficit degree and nitrogen application rate. ** *p* < 0.01 (Duncan’s multiple range test).

**Table 4:** Weights of indicators calculated based on the entropy weight method in 2022 and 2023.

Index	2022			2023		
	e	d	ω	e	d	ω
ET	0.844	0.156	21.78%	0.861	0.139	19.12%
Y	0.890	0.110	15.42%	0.852	0.148	20.33%
WUE	0.841	0.159	22.20%	0.857	0.143	19.67%
NPFP	0.914	0.086	12.04%	0.916	0.084	11.53%
TSS	0.905	0.095	13.32%	0.900	0.100	13.78%
VC	0.891	0.109	15.24%	0.886	0.114	15.58%

Note: ET is total water consumption; Y is yield; WUE is water use efficiency; NPFP is nitrogen fertilizer bias productivity; TSS is soluble solids; VC is vitamin C; e is information entropy; d is the value of information utility; and ω is a weighting factor.

**Table 5:** Combined rankings for each treatment in 2022 and 2023 based on the TOPSIS method.

Index	2022				2023			
	d^+^	d^−^	Ti	Rank	d^+^	d^−^	Ti	Rank
W_1_N_1_	0.717	0.392	0.353	8	0.773	0.326	0.297	8
W_2_N_1_	0.541	0.534	0.497	4	0.550	0.502	0.478	6
W_3_N_1_	0.623	0.556	0.472	5	0.707	0.443	0.385	7
W_1_N_2_	0.655	0.514	0.440	6	0.565	0.533	0.485	4
W_2_N_2_	0.261	0.898	0.775	1	0.179	0.951	0.841	1
W_3_N_2_	0.272	0.805	0.748	2	0.270	0.830	0.754	2
W_1_N_3_	0.971	0.066	0.064	9	0.956	0.121	0.112	9
W_2_N_3_	0.615	0.438	0.416	7	0.571	0.532	0.482	5
W_3_N_3_	0.490	0.658	0.573	3	0.456	0.619	0.576	3

Note: d^+^ and d^−^ denote the distance between the evaluated object and the positive and negative ideal solutions, respectively; Ti is the degree of proximity of the evaluated object to the optimal solution, and a higher Ti indicates closer proximity to the optimal solution.

**Table 6:** Experimental design.

Treatments	Seedling	Flowering and Fruiting	Full Fruiting	Later Fruiting	Fertilizer Rate (kg·ha^−1^)	Irrigation Amount During the Entire Growth Season (mm)	
						2022	2023
W_1_N_1_	70–80%	50–60% ^a^	70–80%	70–80%	215	193	196
W_2_N_1_	70–80%	60–70%	70–80%	70–80%	215	207	217
W_3_N_1_	70–80%	70–80%	70–80%	70–80%	215	238	245
W_1_N_2_	70–80%	50–60%	70–80%	70–80%	270	189	193
W_2_N_2_	70–80%	60–70%	70–80%	70–80%	270	215	228
W_3_N_2_	70–80%	70–80%	70–80%	70–80%	270	250	248
W_1_N_3_	70–80%	50–60%	70–80%	70–80%	325	183	191
W_2_N_3_	70–80%	60–70%	70–80%	70–80%	325	219	216
W_3_N_3_	70–80%	70–80%	70–80%	70–80%	325	254	249
CK	70–80%	70–80%	70–80%	70–80%	0	232	233

^a^ The upper and lower limits of soil water contents (the percentage of field water capacity).

**Table 7:** Division of growth period of eggplant.

Growing Season	Seedling	Flowering and Fruiting	Full Fruiting	Later Fruiting
2022	05.11~06.03	06.04~07.05	07.06~08.02	08.03~08.30
2023	05.14~06.01	06.02~06.30	07.01~07.31	08.01~08.31

## Data Availability

The datasets used and/or analyzed during the current study are available from the corresponding author upon reasonable request and the approval of the data owner.
